# Metallic Dental Implants Wear Mechanisms, Materials, and Manufacturing Processes: A Literature Review

**DOI:** 10.3390/ma16010161

**Published:** 2022-12-24

**Authors:** Sudip Saha, Sougata Roy

**Affiliations:** Department of Mechanical Engineering, University of North Dakota, Grand Forks, ND 58202, USA

**Keywords:** dental implants, metals, mechanical properties, wear, additive manufacturing, tribology

## Abstract

Objectives: From the treatment of damaged teeth to replacing missing teeth, dental biomaterials cover the scientific interest of many fields. Dental biomaterials are one of the implants whose effective life depends vastly on their material and manufacturing techniques. The purpose of this review is to summarize the important aspects for metallic dental implants from biomedical, mechanical and materials science perspectives. The review article will focus on five major aspects as mentioned below. Tooth anatomy: Maximizing the implant performance depends on proper understanding of human tooth anatomy and the failure behavior of the implants. Major parts from tooth anatomy including saliva characteristics are explored in this section. Wear mechanisms: The prominent wear mechanisms having a high impact on dental wear are abrasive, adhesive, fatigue and corrosion wear. To imitate the physiological working condition of dental implants, reports on the broad range of mastication force and various composition of artificial saliva have been included in this section, which can affect the tribo-corrosion behavior of dental implants. Dental implants classifications: The review paper includes a dedicated discussion on major dental implants types and their details for better understanding their applicability and characteristics. Implant materials: As of today, the most established dental implant materials are SS316L, cobalt chrome alloy and titanium. Detailed discussion on their material properties, microstructures, phase transformations and chemical compositions have been discussed here. Manufacturing techniques: In terms of different production methods, the lost wax casting method as traditional manufacturing is considered. Selective Laser Melting (SLM) and Directed Energy Deposition (DED) as additive manufacturing techniques (AM) have been discussed. For AM, the relationships between process–property–performance details have been explored briefly. The effectiveness of different manufacturing techniques was compared based on porosity distribution, mechanical and biomechanical properties. Summary: Despite having substantial research available on dental implants, there is a lack of systematic reviews to present a holistic viewpoint combining state-of-the-art from biomedical, mechanical, materials science and manufacturing perspectives. This review article attempts to combine a wide variety of analyzing approaches from those interdisciplinary fields to deliver deeper insights to researchers both in academia and industry to develop next-generation dental implants.

## 1. Introduction

Human oral health is a significant factor in overall human health. Dental science knowledge is constantly growing by leveraging new science and technologies that are not limited to the medical field, including engineering design, materials, and manufacturing. Many complex mechanical, chemical, and physiological actions are associated with the human food intake system [1]. The combination of these widely varied actions makes predicting their effects on oral condition difficult. Tooth wear is a natural process since teeth are subjected to mechanical and chemical stressors [2,3,4], including damaged or missing teeth, misalignment, and discoloration. These problems can be triggered by interactions between food particles and teeth in the presence of saliva, the nature of the food, and oral hygiene practices. Tooth wear can also be caused by bruxism, overbites or underbites, and missing teeth. A fundamental understanding of these wear mechanisms is necessary to explore a wide variety of dental implants and their materials and manufacturing state of the art. We will discuss dental anatomy and key wear modes along with important wear mechanisms.

Dental implants have existed for more than 2000 years; however, modern dental implant use began during World War II in the form of dental restoration. One of the most important achievements in dentistry occurred in 1957: the Brånemark System. Per-Ingvar Brånemark, a Swedish orthopedic surgeon, discovered the possibility of bone growth in titanium implant proximity, a phenomenon he called ‘osseointegration,’ which has been one of the basic processes used for under-gum implants [5]. The number of dental implants installed per year is between 100,000 and 300,000, according to the American Association of Oral and Maxillofacial Surgeons [6]. Dental implants are a continuously growing market for both medical and cosmetic purposes. Modern science has allowed dentists to offer a wide variety of implants, such as crowns, screws, dental bridges, dentures, and braces [7]. Implant reliability and proper design are essential for patient comfort and usage. The selection of suitable materials, depending on the patient’s oral health and implant objectives, is the primary criteria for proper implant design. Dentistry has progressed significantly since its inception. Implant production has advanced from the use of readily available materials to high-end novel materials. Biocompatibility is the most critical factor when identifying implant materials.

While biocompatibility is the most critical factor when identifying implant materials, other crucial characteristics include toughness, corrosion resistance, fracture resistance and material strength [8]. Numerous studies have been conducted based on these characteristics to evaluate the effectiveness of various implant materials. Saini et al. (2015) [9] presented three implant material categories based on their biodynamic activity: biotolerant, bio-inert, and bioactive. Metal implants made of gold, stainless steel, Co-Cr alloys, niobium, and tantalum are among the biotolerant category. The biotolerant group also includes polymer implants made of polyethylene, polyamide, and polymethylmethacrylate. A very significant metal group, commercially pure titanium and titanium alloys, is part of the bio-inert group. Ceramics made of zirconium oxide and aluminum oxide also belong to the bio-inert category. Ceramics such as carbon–silicon, hydroxyapatite, tricalcium phosphate, and bio-glass are the materials in the bioactive group.

Ongoing research on the biological response to implants as well as clinical observations has decreased the number of usable metals, polymers, and ceramics [10]. Surface topography, roughness, material, and tribological behaviors are also important for the material selection process [11]. The combination of different metals with other ceramics, nutrient elements for the human body, and selecting appropriate coatings to prevent corrosion creates a broad spectrum of research opportunities. Titanium is the most favorable among metals due to its favorable long-term clinical success record, particularly in the case of endosseous dental implants [12,13]. Titanium outperforms other metallic materials in terms of biocompatibility, greater corrosion resistance, and the lack of localized breakdown in numerous corrosion investigations [14,15]. Potential causes of titanium implant failure include high cyclic occlusal loading and hydrogen absorption from the biological environment promoting implant fracture [16,17,18]. On the other hand, ceramic dental prosthesis has been used extensively attributing to their aesthetic benefits and improved material strength [19]. Ceramics also have the advantage of improving bone apposition with the implant by releasing calcium phosphate ions into the area around the implant [20]. However, biomechanical overload can initiate crack propagation in ceramic implants, and due to the brittle nature of ceramics, excessive stress concentration can enhance the crack propagation too, resulting in early implant failure [21,22]. The scope of this review article concentrates primarily on metallic materials and their fabrication routes.

The advancement in dental implants happened due to synergistic effects caused by material and modern manufacturing technique advancement. The dental implant manufacturing process depends on numerous factors, including time, cost, geometrical accuracy, and complexity. Casting is still one of the predominant manufacturing processes in dentistry, dating back to ancient China or Egypt; however, recent advancements in computer-aided technologies and process controls have significantly advanced dental implant design [23,24]. Establishing standardized manufacturing techniques, reducing production costs, and achieving higher accuracies for patient-specific requirements necessitated automated implant production approximately thirty years ago [25]. The implant manufacturing industry is now exploring the additive manufacturing field, since it can be used for exciting new possibilities, such as rapid prototyping, achieving geometrical accuracy with complicated designs, and patient-specific implant model preparation. Achieving higher geometrical accuracy is the primary purpose of the specific manufacturing methods used to fabricate dental implants [26]. The manufacturing process needs to be low cost and with a shorter lead time for delivery. There are pros and cons of both traditional and advanced manufacturing processes. Rapid prototyping can eliminate the waxing step and reduce geometric error [27]; however, it may increase cost.

Post fabrication via different routes, detailed microstructural analysis, suitable material property and performance investigations must be carried out from the application viewpoint of dental prosthesis. Tensile strength, microhardness, three-point bending, and nanoindentation experiments are a few common testing directions carried out in earlier studies [24,28,29,30,31] to evaluate mechanical properties. Common avenues of corrosion resistance behavior of the implant materials include mass decrement analysis [28], open circuit potential (OCP), linear and cycling potentiodynamic polarization and electrochemical impedance spectroscopy (EIS) [32,33,34,35,36]. Another crucial testing involves understanding the tribological behavior of implant materials in artificial saliva. This helps to simulate the appropriate masticatory stress as well as other physiological parameters, providing vital information about implant performance and dependability [28,37,38,39,40].

Alemanno et al. (2020) [18] conducted a comparative analysis focusing on the tribological behavior of titanium alloy and zirconia using artificial saliva as a lubricant. The resulting wear scar area was measured to determine the wear resistance of the alloy in different lubricants; however, more reliable data would be to measure the wear volume with a detailed explanation on the major wear mechanisms for both alloys. In a tribo-corrosion testing of cobalt–chromium alloy under dry and wet conditions, Duran et al. (2019) [38] conducted only an open circuit potential (OCP) test besides traditional wear testing to analyze the corrosive behavior, whereas widely accepted standard corrosion resistance tests for dental implants are potentiodynamic polarization and EIS along with OCP testing. In a review of implant biomaterials by Saini et al. (2015) [39], different mechanical and surface properties of commonly used implant materials were described, but it lacked information on the material microstructure. Similar shortcomings of microstructural evaluation as well as wear mechanism morphology were also noticed in the published work of Zhou and Zheng (2008) [40]. A comprehensive review of metal implants by Rituerto Sin et al. (2013) [41] extensively covers the tribology and tribo-corrosion behavior of metal implants and presents a holistic view. However, discussions on different manufacturing techniques were overlooked, although implant tribology and corrosive behavior changes significantly based on their fabrication techniques.

A dental implant’s success and survival rate depends primarily on its biological and mechanical properties. The biological perspective includes proper osseointegration, correct placement, bone augmentation, and implant toxicity. The mechanical perspective includes implant strength, fracture probability, screw joint instability, and the chance of loosening [41]. It is necessary to conduct a detailed literature review with the proper background knowledge on such interdisciplinary subjects prior to starting experiments on dental prosthetics. The author believes that the information presented in this review paper covers a wide range of topics that are required to select proper fabrication routes with suitable materials and utilize a reasonable process parameter window to characterize the performance of fabricated implants in practical application. Future research can reveal implant mechanics thanks to the thorough explanation of wear mechanisms based on application scenarios. This article’s summary of the various implant materials and production processes can help other researchers narrow down the scope of their work. An in-depth examination of concepts as fundamental as tooth geometry will provide a strong foundation for studies in the field of oral implants. There are few to no comprehensive review articles that focus on concurrent dental prosthetic material and manufacturing techniques with detailed descriptions of popular dental implants, human tooth anatomy, and dominant wear mechanisms to the best of the author’s knowledge [7,9,11,42,43,44,45,46,47,48,49,50,51]. The purpose of this review article is to present a comprehensive review of dental anatomy and wear modes and implants, summarizing fundamental knowledge in this field. We will discuss metallic implant materials and manufacturing techniques, including traditional casting and recent metal additive manufacturing-based fabrication routes. We also present a comparative analysis of additively manufactured versus traditionally fabricated parts to examine the performance and determine future research topics.

## 2. Human Mouth Anatomy

The mouth is often called the oral or buccal cavity. This cavity is the entrance to the digestive system, allowing food and air to enter the body. Cheeks form the lateral walls of the oral cavity, and other components include the teeth, tongue, salivary glands, and ducts [52]. We will be discussing the teeth, teeth roots, and saliva, which are three primary body parts involved in dentistry [52].

### 2.1. Teeth

**Figure 1:**
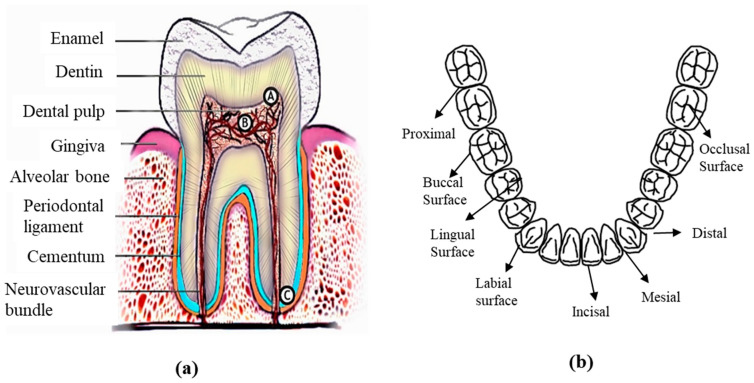
Scientific terminologies related to (**a**) tooth geometry [54] (**b**) tooth surfaces.

Teeth grind and tear ingested food into smaller digestible pieces, which defines the oral cavity’s primary structure and purpose [53]. The hardest and toughest organ in the oral cavity is the tooth. A tooth has two main parts: the crown and root. The root is housed in the bone under the gums, and the dental crown, or covering dentin, is the visible portion of the structure [4] (Figure 1a). Dental tissue is primarily composed of dentin, enamel, cement, and pulp. Important terminology related to the tooth’s surface is depicted in Figure 1b.

Dentin is the uppermost part of the crown, which is covered by highly mineralized protective enamel [55]. The dentin is primarily composed of minerals, with a 70% weight occupying a 40–45% volume. The second component is an organic matrix, with a 20% weight occupying 30% in volume. Water is the last component, with a 10% weight occupying 20–25% in volume [55].

Enamel covers the dentin and is the hardest tissue in the human body. Enamel’s hardness and elastic modulus values range from 3.3 to 5.7 GPa and 72 to 105 GPa, respectively [56,57,58,59,60,61,62,63]. Another study established enamel’s Poisson’s ratio and roughness at 0.28 and 0.20 μm, respectively [3]. Enamel consists of aligned prisms and maintains a perpendicular rod-like shape at the dentin–enamel junction; therefore, it exhibits good wear resistance. This structure provides suitable support for chewing, which is physiologically important. The high mineral content dictates its hardness, and the high elastic modulus and low tensile strength dictate its fragility. The enamel–dentin junction dissipates occlusal stresses and resists impact [4].

Cement and pulp cover dentin at the root surface, ensuring tooth sensitivity. Pulp is the softer, living inner structure that houses blood vessels and nerves.

### 2.2. Root

A specialized calcified substance called cementum is a pale yellow, tough calcium deposit that covers a tooth’s root [55]. The root lies below the cementoenamel junction, is coved with cementum, and connects the tooth to the bone socket. Additional key terms related to the tooth surface that are crucial to comprehend to this work is mentioned below and presented in Figure 1b:

Occlusal: The chewing surface of the posterior teeth.
Distal: The surface facing away from the face’s midline.
Mesial: The surface closest to the face’s midline.
Incisal: The tooth’s biting edge.
Facial: The surface that faces the cheeks or lips. This surface is called labial when it faces the lips and buccal when it faces the cheeks.
Lingual: The surface facing the tongue.
Proximal: The surface between adjacent teeth.

### 2.3. Saliva and Its Composition

Saliva’s influence on food structure modification and lubrication is vital for the dynamic food breakdown process [1,64]. This substance is a prominent factor in food mastication and has several uses: wetting, lubrication, hydration, emulsification, and flocculation [65]. Saliva prevents hard particle abrasion on oral surfaces [66] and forms a protective pellicle protein layer. Saliva’s natural composition is complex: 99% water and various proteins and electrolytes [67]. Researchers have chemically formulated artificial saliva to mimic actual oral cavity interaction scenarios and replicate tooth wear. A concise list of artificial saliva chemical compositions and their respective studies are listed in Table 1.

Saliva may not prevent erosion completely; however, it significantly reduces the likelihood of atherosclerosis [78]. In terms of fluid characteristics, saliva is non-Newtonian and exhibits shear-thinning nature [79]. Saliva is also highly elastic and can adsorb to the oral surface to act as an effective lubricant due to salivary proteins. The saliva secretion rate and viscosity depend on the stimulation type and method [80]. Important tribological benefits include friction reduction between tissues, decreased tooth wear, and tongue surface lubrication.

## 3. Major Dental Wear Types, Locations, and Corresponding Mechanisms

Lack of toxicity, biocompatibility, preventing bacterial microleakage, low plaque formation, and the ability to replace damaged tissue are essential biological qualities for dental materials [81,82,83]. As a functional property, the fracture and wear resistance of dental materials are of major interest [84,85] for long-term reliability, as the primary causes for implant failures are various wear modes [86]. Understanding the reasons and mechanisms underlying tooth wear is crucial for developing functional dental prosthetics. Contact regions between the tooth or any other restorative material and foreign particles in the oral cavity consist of four components [4]: (a) a solid-body representing teeth; (b) counter bodies opposite of teeth or other food elements; (c) interfacial elements between the solid and counter body, usually food products, small particles, saliva as a liquid lubricant, and gas; and (d) the surrounding media or environment, usually air or saliva. One of the most important physiological processes in a tooth’s life is dental wear [2], which is caused by regular mastication force, overbite or underbite, lack of teeth, and bruxism [4].

### 3.1. Dental Wear Mode Types

The definition of wear is “The ultimate consequence of the interaction between surfaces which is manifested in the gradual removal of material” [87]. The most important factor in wear analysis is masticatory force, which is often called occlusal load. Determining the range of this force is difficult, since it varies from person to person with different ages, food habits, and jaw shapes. Researchers have used a range of forces when analyzing appropriate materials for dental prosthetics. Table 2 lists the different mastication forces examined by researchers over time.

Dental wear is a complex phenomenon that is an amalgamation of different simultaneous and sequential mechanisms [78] with four dominant wear mechanisms: (a) abrasive wear (two and three body abrasion), (b) adhesive wear, (c) fatigue wear, and (d) corrosion wear.

### 3.2. Abrasive Wear

**Figure 2:**
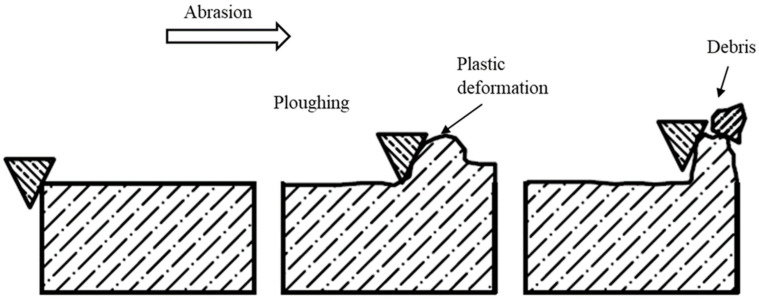
Schematic diagram depicting abrasive wear mechanisms.

Abrasive wear is the most prominent and common wear mechanism observed in human teeth. A common misconception is that only chewing processes result in surface to surface contact; however, involuntary grinding or tooth tightening is also an important initiator. Crucial factors for this wear type include mastication force, tooth condition, bone support, and number of teeth in the oral cavity. Comparatively hard asperities plough into the softer surfaces upon contact with other surfaces, resulting in debris that can act as abrasive particles (Figure 2). Contact material hardness plays a vital role in abrasive wear [87], which is divided into two types depending on the constituents: two-body and three-body abrasion.

#### 3.2.1. Two-Body Abrasion

**Figure 3:**
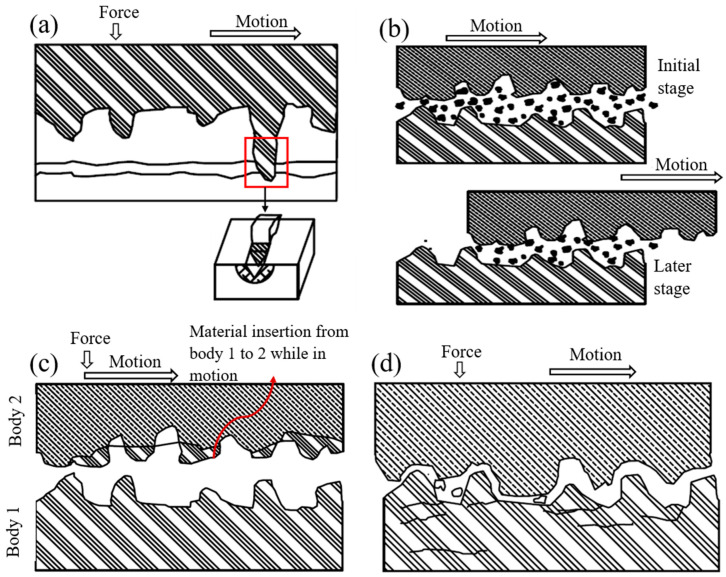
(**a**) Two-body abrasion, (**b**) three-body abrasion, (**c**) adhesive wear, (**d**) fatigue wear mechanism.

Two-body abrasion wear is initiated when two surfaces directly contact each other (Figure 3a). Certain parameters significantly affect two-body abrasion phenomena: sliding distance, contact angle and pressure, attack angle, and sliding speed [93]. A micro-ploughing phenomenon begins when harder surface asperities slide and dig into comparatively softer surfaces. A principal furrow forms along the symmetrical lateral borders. Local deformation or debris formation, also known as micro-fatigue, begins when the groove’s proximity ends due to ductile material weakening. Micro-cutting occurs if there is no plastic distortion on the surface [78]. These microscopic losses can result in visible macroscopic wear when the harder or sharper surface slides over the weaker surface [2]. The friction between the proximal surfaces depends upon two factors: (a) relative lateral motion and (b) closing movement in the chewing process. Relative lateral motion is often termed buccolingual lateral movement. Friction between the medial and distal surfaces acting along the contact point’s perpendicular plane causes wear. The occlusal load, or mastication forces, are essential in the chewing process. This force is distributed on the food bolus, resulting in tearing, breaking, and grinding, making it suitable for swallowing and digestion. Tooth–tooth contact happens with full mastication force upon reaching the food bolus’ full penetration [94].

#### 3.2.2. Three-Body Abrasion

Three-body abrasion occurs when two bodies slide along each other with an external particle interposing between them (Figure 3b). Three-body abrasion can be differentiated into two categories depending on the separation distance between the contact pair surfaces. When the distance between the mandibular and maxillary surface is relatively large, the food bolus or other particles, cumulatively named as intervening particles, act as an overall abrasive suspension over the entire contact region. When the mandibular and maxillary surfaces are much closer to each other, the entrapped particles introduce shearing. These surfaces also move along the adjacent two surfaces and create grooves and fissures [78]. The particles trapped between the upper and lower jaw can continue dental wear even when the occlusal forces are absent, usually during swallowing or cleansing. Hard food elements or small foreign bodies can be integrated into the tooth surfaces due to abrasive force, which can cause severe effects on the three-body abrasion.

### 3.3. Adhesive Wear

Adhesive wear begins when two bodies typically associated with metal and composite restorations slide against each other in the presence of substantial pressure in the oral cavity [4,78]. Material from one surface can be transferred to another due to pressure and sliding motion (Figure 3c). These transferred materials can protrude from dental restorative material or be separate particles enmeshed between the surfaces [87]. Wassell et al. (1994) [95], Söderholm et al. (1998) [96], and Hacker et al. (1996) [97] presented the transfer of restorative materials, such as gold, composites, and amalgam, to enamel during two-body in vitro friction tests [78]. The material transfer from surface to surface was controlled by several factors: contact length, distance between materials, mechanical properties, chemical properties, contact pressure, surface geometry, and physiological environment. These transferred materials break down into debris and cause three-body abrasion when sliding between the surfaces [4,78]. This type of wear is limited to the oral cavity due to the saliva’s physiological lubricating property [78].

### 3.4. Fatigue Wear

Fatigue loading can occur when a surface is subjected to movement under high-pressure and cyclic loading. This wear begins at the surface, and generated micro-cracks can be extended to sub-surface regions (Figure 3d). These micro-cracks then propagate during repeated cycles along sub-surfaces and result in enamel fragmentation in the form of debris. The enamel’s low tensile strength accelerates this fragmentation; however, its prismatic composition hinders micro-crack propagation [98]. These micro-cracks cannot propagate inside dentine because of the enamel–dentin junction; however, this wear can be amplified significantly in an acidic environment and with cyclic loading.

### 3.5. Corrosive Wear

The removal or loss of dental molecules in the presence of a chemical effect is referred to as corrosive wear. Corrosive wear is now one of the dominant forms of wear due to the changes in human eating habits [99]. Corrosive wear may occur due to acid interactions with teeth, resulting in dental tissue intermolecular bond breaking, promoting an increase in other wear mechanisms [100]. A new surface is then exposed due to debris removal and is immediately subjected to the corrosive environment. The cumulative effects of this layer removal at the molecular level result in wearing or cracking on the tooth’s surface. Corrosive agents are non-bacterial and can be classified as extrinsic or intrinsic. Foodstuffs and environmental chemical elements, such as fruits, alcoholic beverages, sodas, or energy drinks, are extrinsic sources. Gastro-regurgitation, disorder-esophagi, and spontaneous vomiting resulting from chronic alcoholism are intrinsic sources [4,78].

## 4. Dental Prosthetics or Implants

Dental implants or prosthetics are used to replace missing teeth and treat partially or fully damaged teeth. The dental implant success rate is more than 90% after ten years, ensuring long-term reliability [101]. There are three primary types of dental implants: endosteal or endosseous, subperiosteal, and transosteal.

### 4.1. Endosteal or Endosseous Dental Implants

**Figure 4:**
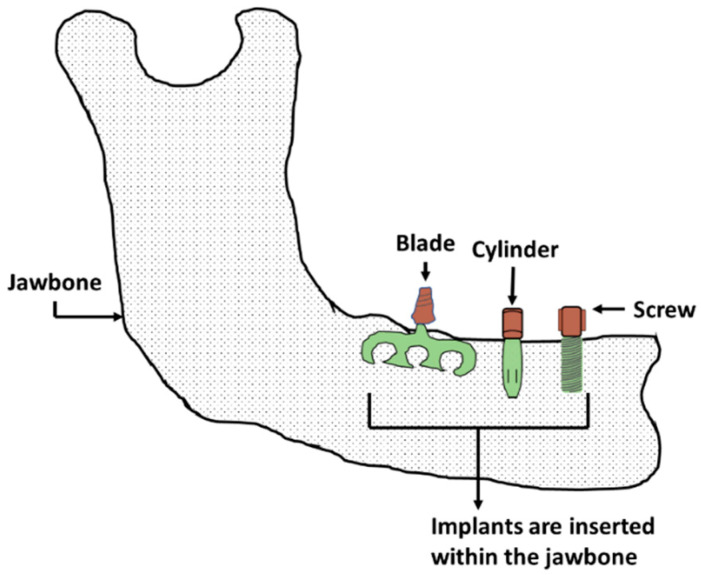
Endosteal or endosseous dental implants.

Endosseous dental implants are inserted within the mandible or maxilla and serve as the dental root (Figure 4). Titanium is typically used as the base material for this kind of implant. Cp (commercially pure) grade 4 titanium is used in many implants; however, titanium alloys are often used because of their higher corrosion resistance, strength, and biocompatibility. Ti-6Al-4V and TiZr are the two primary alloys used [5,11]. There are two types of endosteal implants: observed-root and blade implants.

#### 4.1.1. Root Implants

Endosseous root implants are used to support dentures or replace a damaged tooth’s root. This implant can be inserted into either the mandible or maxilla, depending on the damage location. The type of implant needed depends on the patient’s root cavity condition, damage type, and age. There are three major root implant types: screw threads, solid-body press-fit design, and porous material coated design. The physical topography of this implant may be cylindrical or conical shape (Figure 4). Screw threads or solid body press-fits can be added to a tooth’s primary shape. Most implants are cylindrical, conical, or thread shape, the most common of which is threaded or screw shape (Figure 4). Extensive research has been performed on the factors that improve screw implant success rates, such as implant diameter, implant length, geometry, and threading [102,103,104,105]. A root screw’s length ranges between 8 and 15 mm with diameters ranging between 3 and 7 mm [5]. A longer screw can dissipate stress with a large surface area, which results in good osseointegration. Wider screws can interact with larger bone areas and are better suited for removal torque tests [5,50]. The addition of threads to the root implant aids in improving initial stability, increasing initial contact, and enlarging the surface area. The most common thread designs are V-thread, square thread, and buttress thread. Square threaded designs exhibit more bone compaction and biomechanical integration than others [51,106].

#### 4.1.2. Blade Implants

**Figure 5:**
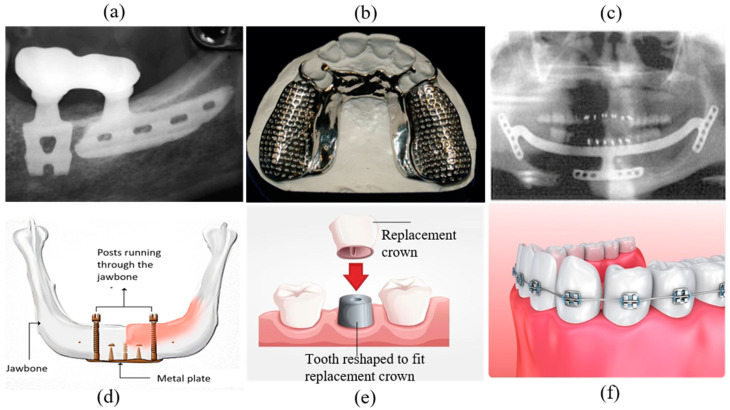
(**a**) Two-blade implant supporting a fixed prosthesis [107], (**b**) a removable partial denture [116], (**c**) ‘U’-shaped ramus frame [117], (**d**) a transosteal implant schematic [110], (**e**) dental crown and its placement over a reshaped tooth [118], (**f**) stainless steel dental brace [119].

A blade implant can be a custom-made anchor for a dental prosthetic (Figure 4). Figure 5a depicts a representative image of a blade implant used in human dentistry. This type of implant supports an abutment using a metal plate placed laterally instead of in the vertical direction of a dental root. A critical factor when designing this implant is allowing the implant to bend so it can be positioned parallel to the curved mandible or maxilla and maintain alignment with the abutment. The blade implant is most useful with knife-edged ridges. The metal–bone contact area for a blade implant is higher than a root implant due to its unique design, promoting better osteointegration. The blade implantation procedure is also simpler than other types of implants [107].

### 4.2. Subperiosteal Dental Implants

Subperiosteal implants are positioned between the jawbone and gum. The implant will become fastened to the jawbone through osseointegration. These types of implants include dentures and ramus frames. Dentures can be classified as fixed dental dentures and removable dentures; then, they can be further classified as partial or full based on placement technique and patient need. Removable partial dentures (RPDs) are currently the most popular implant due to anatomical, physiological and economic conditions, even though they require maxillary or mandibular rehabilitation (Figure 5b) [27]. Dentures are typically created using the lost wax technique; however, 3D scanning and rapid prototyping are becoming popular with advancements in materials and manufacturing. Four general classification outlines, Class I, II, III, and IV, were proposed by Dr. Edward Kennedy for partially edentulous arches [108]. RPD could be used for this treatment depending on the categorized oral conditions.

i. CLASS I: Bilateral Posterior Edentulous Area;
ii. CLASS II: Unilateral Posterior Edentulous Area;
iii. CLASS III: Unilateral or Bilateral Edentulous Area(s) Bounded by Remaining Tooth/Teeth;
iv. CLASS IV: Single Edentulous Area Anterior to Remaining Teeth and Crossing the Midline.

A ramus frame is a U-shaped one-piece mandibular implant (Figure 5c) that can be supported at the wisdom teeth and under the chin. Its specific design, tripoidal shape, provides a unique support structure for the mandibular denture [109]. This type of implant may be used if the required dental implants are missing conventional support placements. This frame can also be useful for reducing discomfort caused by damaged gums, resolving chewing difficulties caused by implants, stabilizing an implant in the correct position, and mitigating speaking challenges.

### 4.3. Transosteal Implants

Endosseous and subperiosteal implants require sufficient bone support from the mandible. A transosteal implant can be used if the patient has severe resorption and jaw damage resulting from failure caused by insufficient implant support (Figure 5d). A metal plate with screws piercing through the jawbone is bolted to the lower mandible to support the metal rods of other implants running from the superior mandible to the inferior border, acting as a horizontal support beam [110]. Both intra- and extra-oral incisions are required to ensure proper stability, which is also referred to as a mandibular staple. Transosteal implants are not used frequently, since they are prohibitively expensive.

### 4.4. Other Dental Implant Types

Other types of implants, which are used for aesthetic purposes or to cover the original implant, exist. These implants are usually designed for temporary use and can be redesigned or relocated based on the patient’s needs.

#### 4.4.1. Dental Crown

One of the most common implants is the dental crown (Figure 5e). A crown, or cap, is generally used to protect a weak tooth, restore a broken tooth, or cover a tooth filling and dental implant. A crown’s support system could be a natural tooth, dental implant, or bone [111]. One essential aspect of a dental crown is its placement technique on the root abutment. The implant abutments connect the prosthetics, a dental crown in this case, to the endosteal implant [112]. The dental crown protects the tooth from further damage since it acts as the tooth’s outer surface. A patient can receive a temporary crown while waiting for a permanent one. Crowns can be made from various materials, including metal, porcelain, ceramic, zirconia, or composite resin [113,114]. 

#### 4.4.2. Dental Braces

Braces are temporary, wire-based implants that attach to teeth to fix alignment or crowding primarily for aesthetic purposes (Figure 5f). Brace positioning requires complex tooth defects and brace positioning knowledge as well as the effects of wire tension. Braces are usually made of stainless steel; however, ceramics are increasing in popularity. Stainless steel is the material best suited for braces; however, nickel ions leached from the steel alloy may cause sensitization or allergic reactions [115].

#### 4.4.3. Dental Veneers

Dental veneers are wafer-thin, custom-made shells of tooth-colored porcelain or resin composites designed to cover a tooth’s front surface to improve appearance. The veneer material’s adhesion to the tooth’s surface gives patients a classically shaped look. Veneers are used for stained teeth, misshapen teeth, substantial gaps between front teeth, or worn teeth.

## 5. Key Metallic Materials Used in Dental Prosthetics

Dental implants have been the most effective treatments for missing tooth replacement. Successful implantation relies on proper material selection. The implant’s function and reliability can vary with a material’s surface property, microstructure, constituting elements, and fabrication technique. The material must be biocompatible, and the implant toughness must mimic the natural body part as much as possible [120].

The selection of appropriate implant material is strongly correlated with the choice of dentists’ and patient’s implant requirement [114]. Metals are the preferred choice for endodontic posts, crown and bridges [121], while the complex forms are created using combinations of noble and non-noble (base) metals [121]. Cobalt–chromium alloy has been extensively used for removable partial dentures as well as substructures for ceramic fused implant framework [42]. Composition-wise, CoCrMo and CoCrW are most common in this application. This alloy can also be used in place of missing tooth as fixed partial dentures [122,123]. Dental wires constructed of beta-titanium and stainless steel are frequently utilized during the treatment phases [124]. Titanium has been used extensively for dental abutment and roots [125]. Adding copper as an alloying element to titanium for the root implant has been proven successful as researchers observed strong biofilm development, preventing bacterial infection and promoting bone formation [126]. Zirconia is also gaining popularity as a crown material due to their high strength, wear compatibility and low cost. Due to their excellent strength [127], wear compatibility [128], and affordability [114], the use of zirconia is increasing as a crown material. Table 3 lists the primary benefits and drawbacks of the three dental prosthesis materials that are most frequently used: cobalt–chromium, stainless steel and titanium alloys.

### 5.1. Cobalt–Chromium Alloys

A cobalt–chromium alloy can be used for prosthetic frames, implants, metal covers under a ceramic crown, or removable partial dentures (RPDs). Comparatively cheaper Co-Cr alloy-based metals are used extensively for dental prosthetics instead of exotic materials. A wide variety of Co-Cr-based alloy composites have been studied by previous researchers (Table 4).

Many countries prefer using Co-Cr alloys for dental applications to avoid the toxic effects of nickel in nickel–chromium alloys (Ni-Cr) [42]. Co contains an FCC structure, which will transform to the hexagonal close-pack (HCP) structure when subjected to slow cooling at 417 °C [156]. Cr increases the strength and corrosion resistance of the alloy by forming a metal carbide. Chromium-rich carbides are M_7_C_3_ and M_23_C_6_, where M could be the constituting alloy metal. Cr-rich alloys can form a hard and brittle sigma phase; therefore, caution is advised when using these materials. Local Cr depletion around the sigma phase can reduce corrosion resistance [42]. Mo has a significant effect on the bulk mechanical properties, and it participates in metal carbide formation due to solid solution strengthening, which can aid in creating an oxidized layer during the production process and assist with ceramic bonding [28].

**Figure 6:**
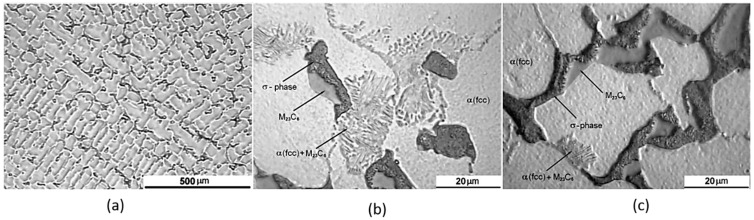
(**a**) Dendritic microstructure of a cast cobalt alloy, (**b**) σ phase and carbide precipitation for an upper cast section, and (**c**) thicker and continuous carbide chain presenting different cooling rates at lower cast sections [157].

Cr-rich alloys can exist in two forms: Co-Cr-Mo and Co-Cr-Mo-W. The former alloy can be used in the casting process, since it is susceptible to reinforcement due to primary molybdenum–carbide formation. The latter alloy creates an oxidized layer during the production process and assists with ceramic bonding [28]. A dendritic α-FCC metastable matrix microstructure is observed in cast Co alloys due to slow FCC-HCP phase transformation. The metal carbides (M_23_C_6_) and sigma (σ) phase can be found spread along the grain boundaries (Figure 6a) and in between the interdendritic spacings. Figure 6b,c depicts the carbide structure and the FCC phase for a cast structure’s upper and bottom sections. The thicker dendrites illustrated in Figure 6c represent different cooling rates depending on the cast structure’s location [157].

### 5.2. Stainless Steels

Stainless steel alloys can be used for ramus frames, dental braces, and crown support. Approximately 60% of the surgical implants used in the United States are made from stainless steel [158]. An AISI 316L-type stainless-steel alloy is widely used for biological implants. The main two constituents for this alloy are Cr and Ni, with weight percentages of approximately 18% and 8%, respectively. The FCC crystal structure ensures shaping and bending flexibility due to its remarkable ductility compared to the BCC ferrite in iron and low-alloy steels [158]. Some typical stainless-steel compositions used in previous dental prosthetics research are listed in Table 5.

Stainless steel alloys are prone to pitting corrosion due to a higher weight percentage of nickel; therefore, caution is advised when using this material for implants, since it can trigger an allergic reaction [9]. Many studies have investigated nickel ion leaching from stainless steel alloy implants in vitro conditions [141,160,161,162,163]. Jensen et al. measured the number of nickel ions released from dental braces based on the nickel content in the oral mucosa cell samples adjacent to the brace and established a release of 0.13 µg nickel ions per cm^2^ after a 1-week immersion in a saliva sample [115].

### 5.3. Titanium-Based Alloys and Comparison to Zirconia-Based Dental Implants

Titanium based alloys can be manufactured with complex shapes based on the individual patient’s needs [143].Titanium based oral implants are currently manufactured from commercially pure titanium or a Ti-6Al-4V alloy (90 wt % titanium, 6 wt % aluminum, 4 wt % vanadium). These alloys have remarkable physical and mechanical properties; however, commercially pure titanium is the most popular choice due to its exceptional corrosion resistance and biocompatibility. Commercially pure titanium is graded into four different types based on purity and oxygen content (Table 6).

The principal alloying component in commercially pure titanium (Cp Ti) is oxygen. The grade and strength of the Cp Ti largely depends on the primary alloying element, oxygen, which strengthens the alloy significantly. With the increasing wt % of oxygen, the tensile and yield strength of titanium also increase. On the contrary, with increasing oxygen amount in the alloy, the % of elongation experiences a downward trend. Cp Ti_4_ has the highest strength of the four grades listed in Table 6 [164].

Pure titanium’s stiffness is not well adapted to the bone, leading to the stress shielding of the residual bone and resulting in detrimental resorptive bone remodeling. A metal’s stiffness is determined by its Young’s modulus and its area moment of inertia. The Young’s modulus for a dental implant is much higher than the cortical bone: the Young’s modulus of pure titanium and the Ti-6Al-4V alloy is 112 and 115 GPa, respectively, while the cortical bone is 10–26 Gpa [165].

Titanium can form solid solutions with atoms that are similar in size. The titanium microstructure consists of hexagonal close pack geometry, or an α structure, up to 882.5 °C. This structure converts to the β structure (BCC) after 882.5 °C until it reaches its melting point of 1688 °C. The alloying elements in titanium play a vital role in determining the microstructure: α, β, or a combination of both. Aluminum or oxygen will function as an α-stabilizer, whereas vanadium, cobalt, tantalum, and iron will function as a β-stabilizer [43]. Ti-6A-l4V, which has an α-β structure, is the most common alloy used for orthodontic applications. The mechanical properties of different titanium structures vary with microstructure. Commercially pure titanium grades have an α structure, unlike Ti-6Al-4V. The Cp Ti grade alloy’s elastic modulus is approximately 102 GPA, but the yield strength varies, with values of 170, 275, 380, and 483 Mpa for grades 1, 2, 3, and 4, respectively. The elastic modulus and yield strength of Ti-6Al-4V are 104 GPA and 795 Mpa, respectively [43].

Titanium and its alloys exhibit excellent corrosion resistance behavior; however, its corrosion resistance property is compromised in the presence of fluoride ions, which can react and form hydrofluoric acid. This acid can destroy the TiO_2_ passive film layer, which can start substantial corrosion propagation, even at low concentrations [143].

Zirconia was used for dental implants approximately three decades earlier than titanium. The primary advantages of zirconia implants are aesthetic value, improved osseointegration, and soft tissue management [9]. Zirconium also does not affect stabilization [43]. Reduction and chlorination reactions turn zirconia into zirconium dioxide (ZrO_2_), which is a polymorphic material that can occur in either monoclinic, tetragonal, or cubic forms, which are only stable at very high temperatures. It is necessary to alloy zirconia with magnesium, yttrium, or calcium oxides to stabilize it at room temperature. Yttrium oxide alloys, also called yttria-stabilized zirconia, stabilize zirconia well. This alloy also possesses sufficient bending strength and high fracture toughness with a high flexural strength of 800–1000 MPa. A zirconia implant can transform into a monoclinic structure from its original tetragonal structure due to aging. This progression begins at the surface and propagates to the core region. The implant’s mechanical properties may degrade due to the domination of the monoclinic phase, resulting in crack initiation on the surface [44]. Gahlert et al. performed a comparative analysis of zirconia and titanium properties, observing that the zirconia sample exhibited a flatter profile with less porosity than the sandblasted and acid etched (SLA) produced titanium sample [166]. A topographic analysis of a zirconia implant produced using SLA exhibited half of the surface roughness of Ti-SLA. Zirconia implants had a mean bone density of 54.6% after 12 weeks of implantation, whereas titanium had a density of 51.6% [166].

Zirconia and titanium have comparable material properties; however, researchers disagree on which is better for dental implants. Zirconia implants can be created to match a patient’s natural teeth, which is aesthetically pleasing and lasts longer than titanium. Titanium implants can cause tissue discoloration or gum graying. Titanium and its alloys may also cause allergic reactions, although this is rare [167]. On the other hand, titanium has been used as a dental implant for several decades; therefore, its design and functionality have been well researched and established. Zirconia has limitations in fabricating implants with complex and customized designs. The long-term success rate for zirconia is yet to be determined.

### 5.4. Noble Metals

Noble metals are a group of metals popular for dental use due to their biocompatibility, physical and mechanical properties, and corrosion resistivity; however, they are expensive due to their unique properties and relative rarity. The noble metal group includes gold, platinum, ruthenium, palladium, iridium, rhodium, silver, and osmium [168]. Noble metal alloy-based dental implants are created using the lost-wax technique. The Council for Scientific Affairs divides prosthodontics alloys into four groups: high noble alloys, noble alloys, predominantly base metals, and Ti and its alloys. The required noble metal content in a high noble alloy must be ≥60%, and the required gold content is ≥40%. The required noble metal content in noble alloys and predominantly base metals is reduced to ≥25%. The required titanium and titanium alloy content must be at least 85% [168]. Gold, palladium, and platinum are the only metals typically used in dentistry.

Gold: Gold is the most widely used noble metal for dental alloys due to its corrosion resistivity, castability, and mechanical properties similar to enamel.

Palladium: Palladium has been used in dentistry instead of gold due to its relatively lower cost; however, current regulations advise against its use as a dental alloy due to its lack of biocompatibility. The PdCu alloy may also cause allergic reactions [169].

Platinum: Platinum is a bluish-white metal with good ductility and malleability. Pure platinum can be used in dentistry because of its high fusing point and good oral condition resistivity. When used with gold, the alloy hardness and elastic quality increases [170].

Noble metal alloys are classified as high noble and noble alloys, and these are listed below from the highest gold content (Au-Pt-Pd-Ag with 78% Au) to lowest (Ag-Pd with 0% Au). The high noble alloys are Au-Pt-Pd-Ag, Au-Cu-Ag-Pd I, Au-Cu-Ag-Pd II, Au-Pt-Pd, and Au-Pd-Ag-In. The noble alloys are Au-Cu-Ag-Pd III, Pd-Cu-Ga, Ag-Pd, Pd-Ag-Au, and Pd-Ga-Au. The alloy’s mechanical and physical properties depend on its elemental composition. Some noble alloys have higher amounts of silver and palladium, which reduce the gold amount and overall density, resulting in castability reduction [168].

## 6. Fabrication Techniques of Dental Prosthetics

A dental implant’s fabrication method significantly affects its performance and reliability, including implant life, osseointegration, and sustainability in a corrosive environment. These manufacturing techniques can be classified into traditional and additive manufacturing.

### 6.1. Traditional Manufacturing Techniques

Casting is the most popular traditional manufacturing technique used to fabricate dental implants. This technique is one of the oldest manufacturing processes, and it is still being used extensively. The casting method can produce complex geometries with acceptable tolerance at a low cost. Lost wax, or investment casting, is the most commonly used fabrication method for delicate implants.

#### Lost Wax Method

The lost wax method includes creating a lower and upper rubber or silicone mold held together with pins. The mold is created from a master model with a sprue, into which molten wax is pumped or injected. The textures and delicate designs become imprinted onto the wax model during solidification.

The dry wax pattern is positioned onto a larger mold with several other wax patterns after the initial mold is removed, creating a tree with pattern branches. The material for this mold may vary according to the different metal fabrication techniques used. The wax patterns are then burned out using steam, a furnace, or another heating source, leaving a cavity for the target metal after the pattern tree is completed [28,171].

The American National Standards Institute classifies casting alloys according to their minimum elongation property and yield strength. Alloys are classified as Types 1, 2, 3, and 4. Low and moderate strength castings are classified as 1 or 2 since they can be easily burnished; however, these castings have limited force resistance. Type 3 castings are the most popular material due to their machinability and surface treatment scope. Type 4 alloys exhibit high strength and can be used for veneer crowns and long-span partial dentures [168].

**Figure 7:**
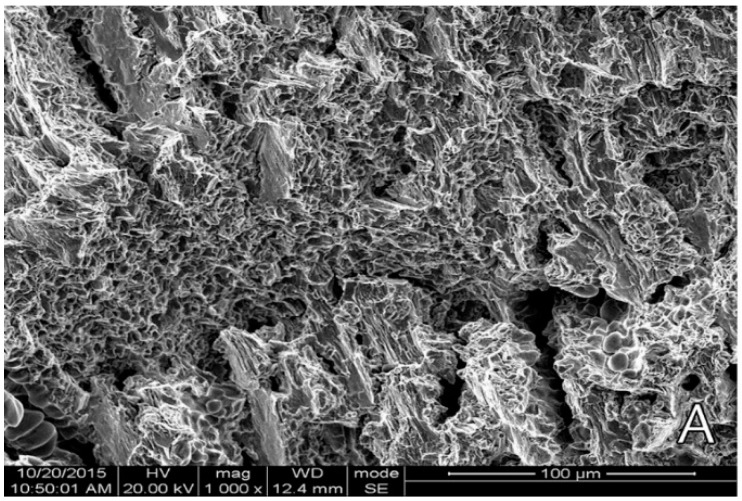
Fracture surface of a cast cobalt–chromium alloy tensile specimen showing cleavage fracture pattern [173].

**Figure 8:**
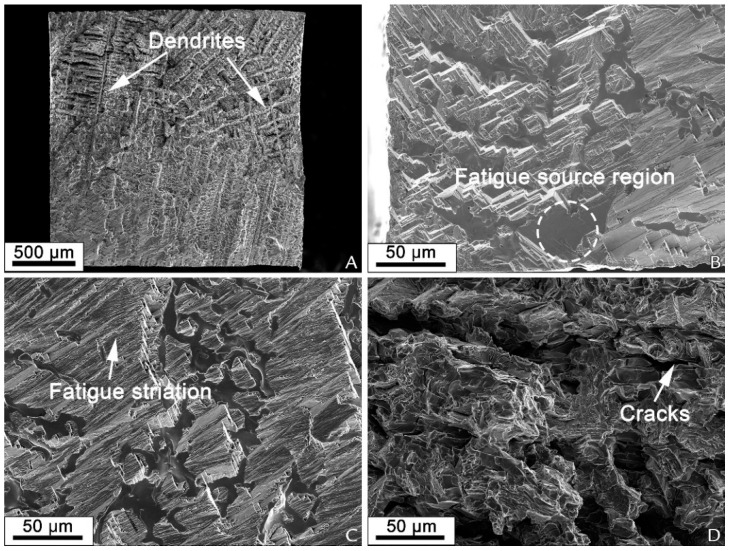
Fracture surfaces of cast cobalt–chromium alloy fatigue specimens showing (**A**) whole fatigue fracture surface, (**B**) fatigue crack initiation region, (**C**) crack propagation region, and (**D**) definitive transient region [172].

Cast cobalt–chrome alloy generally shows a dendritic microstructure as reported [137,172,173]. Analyzing fracture surface from tensile test, Zhou et al. (2017) [173] reported a cleavage fracture pattern (Figure 7) for cast cobalt–chrome alloy with wedge-type cracks forming tearing edges. Wu et al. [172] stated that the interlacing and staggered cleavage planes distribution can initiate a fatigue source region under stress concentration (Figure 8). Kim et al. [153] conducted tensile tests on CoCr alloys prepared from casting and milling according to ISO 22674 and reported the cast and milled groups exhibited deep furrows randomly distributed in the brittle cleave, indicating a brittle fracture pattern.

Augustyn-Pieniazek et al. (2015) [28] conducted a tribological test on four commercially available cobalt–chrome samples (Remanium 2001, Wironit LA, Colado CC, Heraenium P) manufactured using the lost-wax method under an artificial saliva-lubricated condition and reported that the shift in wear mechanism from micro-ridging to micro-scratching was due to the increased test duration.

### 6.2. Additive Manufacturing

**Figure 9:**
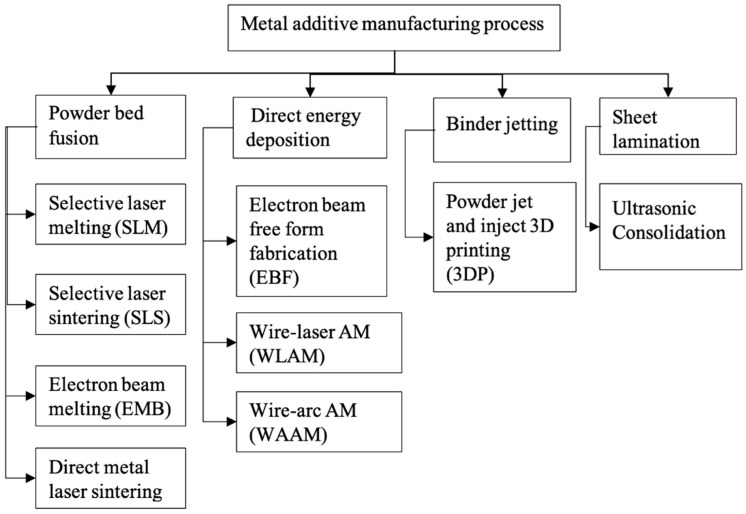
Metal additive manufacturing process classification [174].

The field of additive manufacturing has evolved significantly in the last decade, especially concerning rapid prototyping and achieving dimensional accuracy. Numerous methods are used to classify additive manufacturing processes based on heat source, feedstock supply technique, and melting and binding mechanisms. The metal AM technique can be classified into four basic processes based on the fabrication method: powder bed fusion, direct energy deposition, binder jetting and sheet lamination (Figure 9) [174]. The feedstock material can be in powder, wire, or sheet metal forms. Metal powder has been the most popular feedstock for biomedical prosthetics-based investigations.

Spherical-shaped powder particles produce better flowability and density results. Fine particles positively affect density; however, they can become segregated from grainier particles if not fused properly [175]. The two well-accepted metal AM processes used to develop various biomedical implants are SLM-type powder bed fusion and direct energy deposition.

#### 6.2.1. Selective Laser Melting (SLM)

SLM is one of the most widely studied additive manufacturing processes for printing metal parts. Metal powder rests on a platform that can be lowered according to the preset layer height. A laser beam focuses and scans the powder, resulting in the melting of the scanned powder due to the laser’s heat, after which the remaining powder is removed. The powder bed is then cleared and lowered for subsequent full powder layer sprayings.

An SLM product’s quality depends primarily on two properties: porosity and part density. These properties are greatly influenced by energy density input. A low energy input causes an imperfect molten layer, resulting in large pores. A higher energy input is recommended for higher part density. Vandenbroucke and Kuth investigated fabricating dental parts using biocompatible Ti-6Al-4V metal powders and an SLM-based AM technique; then, they compared the data to an annealed sample. SLM-optimized process parameters with energy densities ranging from 100 to 300 J/mm^3^ could produce maximum part densities of 99.98%. The micro and macrohardness values of the SLM-fabricated Ti-6Al-4V samples were 410 and 400 HV, respectively, whereas the annealed sample value was 350. The ultimate tensile strength for the SLM-printed part was 1250 MPa compared to 1000 MPa for the annealed Ti-6Al-4V. The ultimate bending strength of the SLM-printed part, 2000 MPa, was higher than the annealed part, at 1900 MPa [176]. An SLM product’s microhardness can be higher than its macrohardness due to the high probability of pore-free testing or rapid cooling.

Four different melting zones can be observed as a function of the SLM processes’ scanning speed and laser power, which can be adjusted to properly melt the powders, resulting in a near net dense product. These zones are categorized as Zones I, II, III, and IV, indicating fully dense, over melting, incomplete melting, and overheating regions, respectively [177].

There are very few peer-reviewed studies investigating the corrosion behavior of SLM-printed samples used for dental prosthetics-based applications. Zhang et al. [143] analyzed the corrosion behavior of SLM printed and wrought Ti-6Al-4V. Corrosion tests were performed using artificial saliva as a test solution, revealing that the SLM sample had lower corrosion resistance than the wrought sample. The staircase effect and slope angle greatly influence surface roughness in any AM procedure. An increased sloping angle causes a reduction in the stair effect, which decreases the surface roughness. An increasing sloping angle layer results in decreased thickness, increasing the number of steps [176].

**Figure 10:**
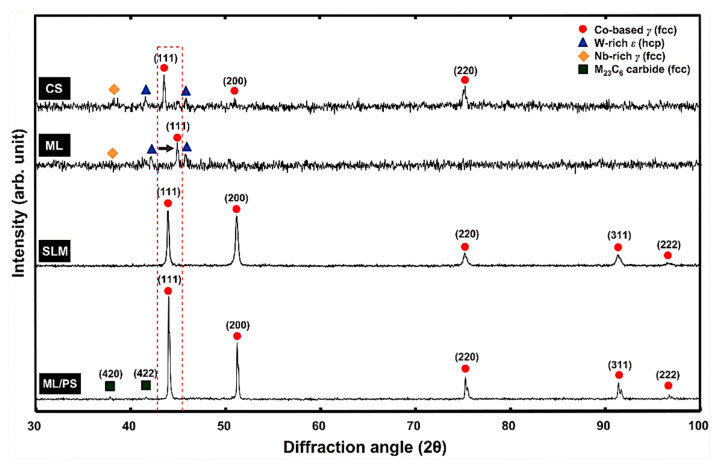
CoCr alloy exhibiting the dominating γ-FCC phase with different manufacturing processes [153].

On the contrary, decreasing layer thickness will increase the printing time and fabrication cost. Vandenbroucke and Kruth analyzed the effects of surface roughness and slope angle on the SLM-printed biocompatible metal alloy Ti-6Al-4V, establishing that the initial surface roughness decreases with the increased slope angle. The surface roughness improvement was negligible upon reaching a high sloping angle of >75° [176]. Kim et al. investigated the microstructure and mechanical properties of Co-Cr dental alloys fabricated using SLM and three other conventional manufacturing techniques, including casting, milling, and milling/post-sintering [153]. Figure 10 depicts the XRD patterns from the Co-Cr alloy sample. All four fabricated samples contained the Co-based matrix of the γ (face-centered cubic) phase. Samples were prepared using casting, and the milling contained the tungsten-rich ε (hexagonal close-packed) phase. The as-fabricated SLM-printed samples did not contain the γ phase in their microstructures.

**Figure 11:**
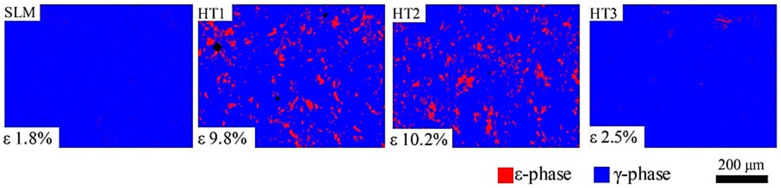
Phase distribution of the CoCr sample at different temperatures, exhibiting an increased ε phase and an increase in heating stage number [30].

One of the primary challenges with the SLM process is the development of high residual stress in the microstructure due to rapid melting and cooling, resulting in compromised reliability with microcrack formation or distortion in the biomedical implants. Li et al. [30] studied the effects of different heat treatment routes on SLM-printed Co-Cr-Mo-based biomedical grade samples as presented in Figure 11. The authors developed three heat treatment routes: HT1, HT2, and HT3. The HT1 samples were heated to 450 °C with a 45 min hold time, then reheated to 750 °C with a 60 min hold time. The HT2 samples were additionally aged at 450 °C for 100 min. The HT3 samples were annealed at 1150 °C for 120 min. The authors observed that post-fabrication heat treatment is a promising process for limiting high residual stress and can increase the ε phase (HCP) percentage up to 10% depending on the heat treatment scheme. The as-printed SLM samples contained 1.8% of the ε phase based on the test samples’ electron backscatter diffraction (EBSD) analysis. The calculated ε phase fractions for the HT2 and HT3 samples were 9.8% and 10.2%, respectively, whereas aging the samples at high temperature, 1150 °C, resulted in homogenized recrystallization with a limited ε phase (2.5%) that was free of residual stress, resulting in a sharp decrease in nanohardness and scratch resistance.

The SLM-printed samples’ hardness increased due to the HT1 and HT2 heat treatment schemes, but it decreased significantly after the HT3 heat treatment. A nanoindentation-based investigation showed the hardness values for the ɣ and ε phases as 5.12 GPa and 6.26 GPa, respectively [30]. The hardness also varied with the build heights of AM samples. Higher hardness values were observed for the samples extracted from substrate vicinity regions due to different cooling rates. The residual depth analysis linked the tribological property to 3D-printing building direction. The longitudinal cross-section exhibited better tribological properties in terms of lower frictional coefficients parallel to the building direction [30]. The mechanical properties of another popular cobalt chrome alloy, F75 fabricated via the SLM technique, were investigated for removable partial denture (RPD) applications [24]. The tensile testing on these SLM printed samples revealed yield and tensile strengths of 689.24 MPa and 1.38 GPa, respectively. These tensile test numbers exceeded the mechanical properties of Remanium GM 380+, which is a commonly used dental material [24].

#### 6.2.2. Directed Energy Deposition

**Figure 12:**
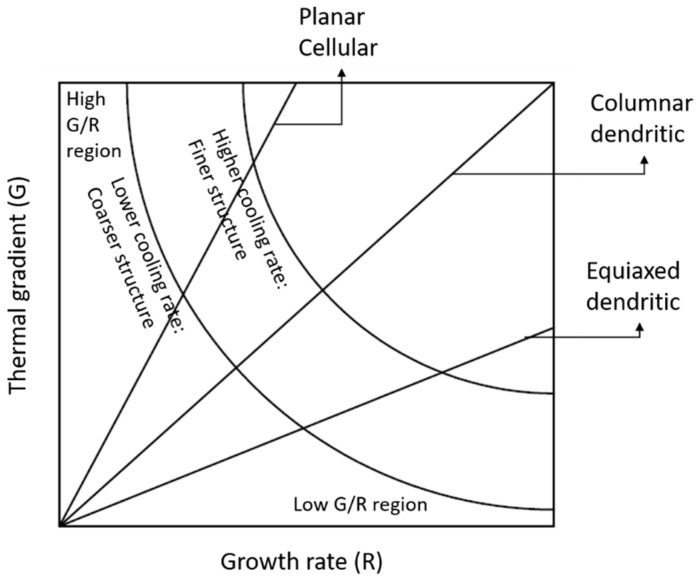
Effect of cooling rate on solidification microstructure related to thermal gradient and growth rate [182].

The directed energy deposition (DED) process uses simultaneous feedstock materials in the form of wire or powder, which are directed to the melt pool location generated by a laser, arc, or electron beam. DED does not require a vacuum, unlike other printing systems such as electron beam melting (E-PBF), and the deposition process happens in the presence of inert gases [178]. A DED setup can be equipped with single or multiple nozzles to deliver metal powders for multimaterial part fabrication. Multiple nozzle setups can be leveraged for functionally graded material fabrication [179]. Macro or micro-porosity is much lower in DED manufactured products if the process parameters are optimized properly, which is advantageous compared to the SLM process [180]. Mathoho et al. [181] analyzed the effects of laser power and scanning speed on porosity formation in 17-4 PH stainless steel using the DED process. Material porosity increased with increasing laser power at a fixed scan speed, which was attributed to increased evaporation due to the usage of high laser power. The DED process melt pool variables significantly affect the final deposit characteristics. The melt pool’s peak temperature is strongly correlated to laser scanning speed and laser power. Experimental studies have determined that increased laser scan speed results in an energy density drop, significantly reducing the melt pool peak temperature. Melt pool temperature mapping can be beneficial when optimizing deposition process parameters. A higher cooling rate is generally observed at the top instead of the bottom surface with a fixed scan speed [182,183]. An additive manufactured product’s material microstructure depends on solidification rate (R) and temperature gradient (G). Figure 12 presents the effects of G and R on the solidification microstructure [182]. Combining these properties allows us to obtain two governing factors for the solidification microstructure: cooling rate (G × R), which governs the solidification microstructure, and G/R, which governs the solidification mode [184]. Finer microstructures are the result of a higher cooling rate, whereas the G/R ratio governs the microstructure’s morphology on the 3D-printed materials: cellular, columnar, or dendritic. These observations are consistent with previous studies [183,185].

Atom diffusion and grain coarsening occur due to a lower cooling rate since the material has more time to solidify and more carbide formation occurs [186,187]. The cooling rate depends on bead depth and scanning speed, decreasing with decreased scan speed and increased bead depth [188]. Tan et al. developed a multiscale model to study the transport phenomena and dendritic growth in laser cladding [189]. A higher cooling rate was observed toward the top of the build direction; therefore, dendritic arm spacing reduced with an increased cooling rate. The effect of cooling rates, between 450 and 2350 °C/s, on SS 316L is presented in Table 7.

**Figure 13:**
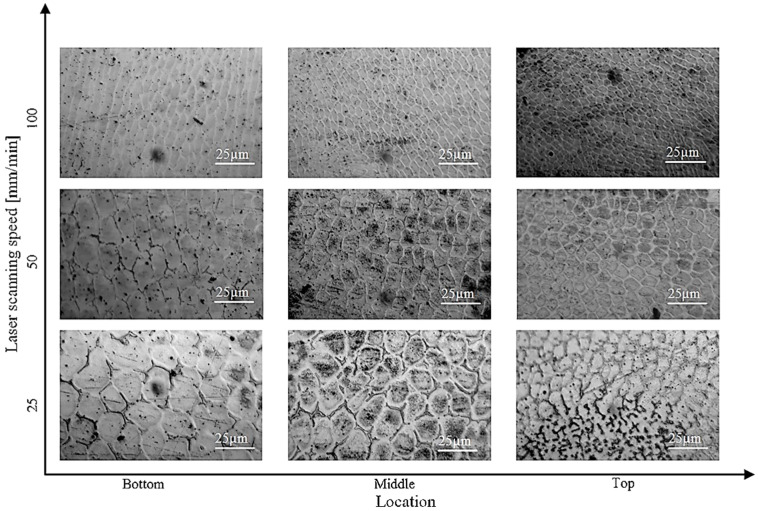
Evolution of as-printed SS 316L sample microstructures with varying scan speeds and at different build height locations [182].

The scanning electron micrograph images in Figure 13 represent the evolution of microstructures in different locations along the build height as a function of laser scanning speed [182]. Reduced primary dendrite arm spacing (PDAS) or fine microstructure can be observed in the top region of the SS 316L due to the increased laser scanning speed. PDAS decreased from 16 to 5.5 µm with scanning speeds between 20 and 40 mm/min.

### 6.3. Comparison between Different Manufacturing Methods

Suleiman and Vult von Steyern et al. [190] compared cast, milled, laser sintered, and high gold cast alloys to showcase the effects of manufacturing methods on the fracture strength of porcelain fused Co-Cr crown samples. The high cast gold alloy control group had the highest fracture strength of the manufactured Co-Cr groups. The highest mean fracture strength among the Co-Cr alloys was for the milled group (1643 ± 153 N), followed by cast and laser-sintered alloys, which had similar values of 1560 to 1562.

Myszka and Skrodzki compared the microstructure of Co-Cr dental implants manufactured from SLM and cast with a 3D-printed pattern [191]. The SLM samples were finer-grained and more homogeneous than cast samples; however, the surface roughness was higher for the SLM-printed Co-Cr dental alloy, suggesting that suitable postprocessing is required after fabricating parts using AM to reduce surface roughness. Roughness values for the SLM-printed Co-Cr-Mo-W alloys were higher than the samples created by the investment casting method. This issue can be addressed with the blast cleaning surface processing technique. Lowering the implant metal surface roughness is essential in dentistry to ensure smooth metal–ceramic bonds.

Previous research studies have determined that cast and milled frameworks made from Co-Cr alloys had more pores than samples produced by SLM, resulting in inhomogeneities. These alloys are at risk of having weaker areas and lowering the possibility of clinical success since they lack sufficient homogeneity [192,193]. Barro et al. [180] investigated the comparative performance of laser-directed energy deposition process (LDED) cp-Ti (commercially pure titanium) to milled samples. Microstructural observation revealed serrated colonies for LDED samples due to higher cooling rates and equiaxed structure in milled samples [194]. Finer grain boundaries and grain nucleation were found in the serrated colonies, which improved part homogeneity [195]. LDED exhibited better mechanical properties, supporting the theory that serrated colonies have greater mechanical properties than the equiaxed grain structure [180].

The XRD and EBSD analyses revealed an α-Ti structure (HCP) in the LDED and milled samples. A 7% increase in ultimate tensile strength and 16.7% increase in hardness value was observed for the LDED samples compared to the milled group. Other mechanical properties, such as yield strength and Young’s modulus, were approximately the same. The LDED samples also exhibited an almost 30% increase in toughness modulus compared to milled titanium.

Barbin et al. [196] presented a comparative analysis of a Ti-6Al-4V alloy manufactured using EBM, SLM, and milling (control group) for full-arch dental prostheses (FAFDPs). This research focused on biomechanical behavior more than traditional mechanical property analysis, and screw loosening torque was measured. Screws were initially tightened with a 10 Ncm torque two times with 10 min intervals for screw stability analysis. The screw-loosening torque values were measured after 24 h. This experiment indicated that the milling group required the highest screw loosening torque, which was followed by EBM and SLM.

A screw-loosening torque analysis was also completed after a chewing simulation of 10^6^ mechanical cycles [196] (Mechanical Fatigue Simulator ERIOS, model ER11000 Plus, Sao Paulo, Brazil). There was a similar screw-loosening torque for both milling and EBM, while SLM required lower torque. Burguete et al. [197] determined that a metal framework with a higher surface roughness requires a lower screw-loosening torque. Better fitting between the framework and prosthetic screw can be ensured by having lower surface roughness. The additively manufactured parts had a higher surface roughness. SLM-fabricated samples had the highest tensile strength, which was followed by the casting and milling groups. SEM images depicted particular brittle cleavage after the tensile test, referring to the brittleness in the cast and mill group samples [153].

Almufleh et al. [116] conducted a clinical trial to determine outpatient satisfaction levels for RPD cast and laser-sintered prosthetics using a double-blind test with nine participants. Five participants preferred the laser sintered RPD, one participant preferred cast RPD, and the last three had no preference. Participants expressed higher satisfaction with laser-sintered prosthetics in terms of cleaning, comfort, and oral condition at the end of the trial. Xiang et al. [88] compared the wear properties of Ti-6Al-4V manufactured from EBM to the wrought process. The EBM sample had a finer microstructure than the wrought Ti-6Al-4V. The EBM samples had a lower frictional coefficient than the wrought sample in both lubricated and dry conditions, since the EBM sample had a higher contact angle than the wrought sample. Both sample types experienced adhesive and abrasive wear during the dry friction tests, whereas the abrasive wear was dominant in lubricated conditions. The Ti-6Al-4V-Zr0_2_ tribo-pair exhibits lower wear in terms of depth and volume compared to the Ti-6Al-4V-Al_2_O_3_ pair.

For the successful development of dental implant materials, performance evaluations are carried out utilizing tribological behavior analysis of the materials under proper contact and lubrication conditions. Table 8 presents a short summary of wear performance of different implant materials reported by various researchers based on the manufacturing technique and used process meters during tribological investigations.

## 7. Conclusions and Future Research Directions

The primary goal of this review paper was to establish a link between basic dental anatomy, dental implant types, failure modes, and manufacturing technologies. Specific types of implants need to be designed based on the type of dental injury. A detailed implant analysis must be conducted for root and dentin injuries. Implants include several types of crowns, their support systems, root screws to hold the abutment, and screw types. Implant performance depends on the patient’s physiological conditions. The nature of the mouth cavity changes depending on food type, time of day, physical condition, and saliva, all of which can affect the implant. Preparing artificial saliva for in vitro studies is an integral part of dental research. Medical research does not always cover mechanical and materials science viewpoints, such as microstructural characteristics and tribological analysis on implants and fracture behavior; therefore, it is vital to establish an appropriate link between these interdisciplinary fields to develop next-generation dental implants. The overall process characteristics are poorly known; therefore, a proper understanding of implant materials, their corrosion, wear behavior in body conditions, and in-depth micrographic analyses are crucial factors. The two most prominent wear types observed in various dental implants are adhesive and abrasive wear. These wear types damage the tooth’s surface, eventually requiring crown replacement and, in severe cases, root implants. Surface roughness, mechanical strength, ductility, and hardness should be analyzed when selecting the implant’s material. Any material used as an implant must be biocompatible and non-toxic. Cobalt–chrome alloys, titanium, and stainless steel are the most common materials used for dental implant fabrication. We have thoroughly discussed implant material surface characteristics and mechanical behaviors. The bacterial and corrosion studies of the implant material were not included in this review. This review article does not have significant details on ceramic implants or metal implants with ceramic coating which are becoming popular nowadays thanks to their suitable mechanical property and aesthetic values. For future work, bacterial and corrosion studies can be explored along with detailed ceramic implants specially understanding their fracture modes.

Mechanical properties can depend on different manufacturing methods and surface treatments. Traditional manufacturing methods, such as casting, are still used extensively due to process simplicity and established technological development; however, metal additive manufacturing is the future of the production process due to the minimized human error, reduced defects, enhanced customizability, and higher geometrical accuracy. The selective laser melting (SLM) process has been widely explored for dental implant fabrication compared to other metal AM techniques. Directed energy deposition (DED) has significant potential to successfully print dental implants with enhanced functionality via functionally graded implant developments combining select materials for surface and core regions.

## Figures and Tables

**Table 1:** General artificial saliva compositions used in previous research.

Artificial Saliva Constituents (Weight (g) % in 1 L Water)							References
NaCl	KCl	CaCl_2_	NaH_2_PO_4_	Na_2_S	Urea	Others	
0.4	0.4	0.795	0.78	0.005	1		[3]
0.4	0.4	0.906	0.690	0.005	1		[68]
0.4	0.4	0.795	0.78	0.005	1		[69]
0.4	0.4	0.795	0.69	0.005	1		[70]
0.4	0.4	0.795	0.78	0.005	1		[71]
0.4	0.4	0.795	0.69	0.005	1	KSCN: 0.3	[72]
0.7	0.4	0.795			0.13	Na_2_HPO_4_: 0.19, NaHCO_3_: 0.15, KSCN: 0.33, KH_2_PO_4_: 0.26	[73]
0.7	1.2				1.3	Na_2_HPO_4_: 0.26, NaHCO_3_: 1.5, KSCN: 0.33	[74]
0.7	1.2					Na_2_HPO_4_: 0.26, NaHCO_3_: 1.5, KSCN: 0.33, KH_2_PO_4_: 0.20	[28,46]
0.4	0.4	0.6	0.58		1		[75]
0.3	1.12	0.17				MgCl_2_: 0.06, NaHCO_3_: 0.63, K_2_HPO_4_: 1.5,	[76]
	0.62	0.17				MgCl_2_: 0.059, K_2_HPO_4_: 0.326, C_8_H_15_NaO_8_: 10, C_8_H_8_O_3_: 2	[77]

**Table 2:** Masticatory force ranges measured in previous research.

Tested Materials	Masticatory Force	References
Flat enamel (Human tooth)	20 N	[3]
Zirconia ceramic, Gold-Pt alloy, Human tooth	4 N	[71]
CoCr alloy	22.24 N	[28]
Ti-6Al-4V	3 N	[88]
Commercially pure titanium	20 N	[68]
Cusp and polished flat (Human tooth)	1.9, 3.9, 5.88 N	[89]
Human tooth	20 N	[69]
Resin-based composite	0.98 N	[90]
Cusp and flat (Human tooth)	2, 4, 8 N	[91]
Flat enamel (Human tooth)	30 N	[92]
Cast Ti specimen	5 N	[75]

**Table 3:** Major advantages and disadvantages of most common implant materials used in dental prosthesis.

Metal and Alloys	Advantages	Disadvantages
Cobalt–chromium alloy	Non-magnetic, stain and heat resistant [42] High strength [42,129,130,131] Excellent biocompatibility [132,133] Corrosion resistant [131,134] Wear resistant [135,136]	Cobalt element can result in the greatest release of harmful ions [137]. May cause inflammatory reactions and hyperplasia [138,139].
Stainless steel alloy	High availability Cost effective Formation of passive chromium oxide film resulting corrosion resistance [140]	Nickel and chromium from the alloy can cause hypersensitivity in some people [141]. Can be susceptible to pitting corrosion due to inclusion of dissimilar material in manufacturing process [142].
Titanium and its alloys	Appropriate young modulus High biocompatibility Superior corrosion resistance [143,144,145]	Compromise in corrosion resistance in environment containing fluoride ions [146,147,148,149]. Dissolution of aluminum and vanadium ions can have toxic effects on the host tissue [150,151,152].

**Table 4:** Chemical composition of Co-Cr alloys commonly used for dental prosthetics.

Trade Name	Chemical Composition (wt %)						References
	Co	Cr	Mo	W	Si	Others	
Remanium 2001	63	23	7.3	4.3	1.6	Mn < 1, N < 1	[28]
Wirnoit LA	63.4	29	5	-	1.2		
Colado CC	59	25.5	5.5	5	-		
Heraenium P	59	25	4	10	1		
MTI China	Bal	25.7	5.9	5.6	<1.5	C < 0.03, O < 0.05	[29]
Remanium GM 380+	64.6	29	4.5	-	<1	C < 1, Mn < 1, N < 1	[24]
Sandvik Osprey F75	Bal	27–30	5–7	-	<1	C < 0.35, Mn < 1, Fe < 0.75, Ni < 0.5	
Wironium plus	62.5	29.5	5.0		<1	C < 1, Mn < 1, N < 1, Fe < 1	[42]
Wironit LA	63.5	29.0	5.0		1.2	C < 1, Mn < 1, N < 1	
Brealloy F400	64.7	29.0	5.0		0.5	C: 0.4, Mn: 0.4	
Bego, German	63.9	24.7	5.0	5.4	1.0		[30]
Soft Metal™	53	29	6	10	<1	Fe < 0.1	[153]
Stellite 6	Bal	28	-	4.5	0.9	C: 1, Mn: 1, Fe: 3. Others < 3	[31]
Stellite 6	Bal	31	-	3	-	C: 1.3, Fe: 1.21, P: 0.42, Mn: 0.22	[154]
Stellite 6	Bal	30.2	0.7	4.5	1.0	C: 1.1, Mn: 1.2, Ni: 1.9, Fe: 0.9, *p* < 0.005, S < 0.005	[155]

**Table 5:** Chemical composition of stainless-steel alloys commonly used for analysis.

Metals	Chemical Composition (wt %)						References
	Fe	Cr	Ni	C	Mo	Others	
SS 316L	Balance	16.5–18.5	11.0–14	<0.03	2–25	Si < 1, Mn < 2, *p* < 0.045, S < 0.03	[115]
SS 305	Balance	17–19	11.0–13	<0.07		Si < 1, Mn < 2, *p* < 0.045, S < 0.03	
SS 18/8	73.75	18	8	0.25			[32]
SS 316L	Balance	18	12	<0.03	2.5		
SS 316L (ASTM F-1982)	Balance	17–20	12–14	<0.03	2–4	Mn < 2, *p* < 0.045, S < 0.03, Si < 0.75, N_2_ < 0.1, Cu < 0.5	[159]
SS 316L (ASTM F138-1986)	Balance	17–19	13–15.5	<0.03	2–3	Mn < 2, *p* < 0.025, S < 0.010, Si < 0.75	
SS 316L	Balance	16.64	10.355	0.024	2.037	Mn: 1.519, P: 0.029, S: 0.025, Si: 0.407, Ti: 0.006, N: 0.047, Cu: 0.296, Co: 0.188	[33]

**Table 6:** Commonly used titanium alloy chemical compositions [43].

Material		Ti	Fe	O_2_	N	H_2_	C
Commercially Pure Titanium (Cp Ti)	Grade 1	99%	0.2%	0.18%	0.03%	0.15%	0.1%
	Grade 2		0.2%	0.25	0.03%	0.15%	0.1%
	Grade 3		0.2%	0.35%	0.05%	0.15%	0.1%
	Grade 4		0.3%	0.4%	0.05%	0.15%	0.1%
Ti-6Al-4V		90%	0.25%	0.2%	-	-	-

**Table 7:** Dendritic arm spacing as a function of cooling rate [189].

Approximate Cooling Rate (°C/s)	Approximate Dendritic Arm Spacing (µm)
450–550	10.5
1050–1150	9.4
1400–1600	8
2250–2350	7

**Table 8:** Tribological studies on commonly used metallic implant materials for dental prosthesis.

Material Used	Manufacturing Process	Tribological Experimental Parameters	Wear Mechanisms	References
Co-Cr alloy	Casting, and SLM	Ball-on-disc tribo experiment under dry condition. Used load: 5 N Rotational speed: 200 rpm	Primary wear mechanism was abrasive and fatigue. Plastic deformation was lower for SLM sample and showed overall higher wear resistance.	[136]
Co-Cr alloy (Remanium 2001, Wironit LA, Colado CC, Heraenium P)	Casting	Abrasive wear test on Miller apparatus under SiC and artificial saliva solution condition. Used load: 22.24 N Frequency: 48 rpm	Prominent wear mechanism was micro-scratching with a minor degree of micro-ridging.	[28]
Co-Cr-Mo alloy	Casting and SLM process	Reciprocating sliding wear test in dry and artificial saliva lubricated condition. Used load: 5 N Sliding speed: 1.7 cm/s	Higher wear rate in cast sample due to tendency of hard carbides leaving the matrix. Formation of micro-cracks was observed in SLM-processed sample under wet condition	[198]
Ti-6Al-4V	Wrought and EBM process	Ball-on-disc reciprocating sliding experiment under dry and lubricated (simulated body fluid) condition. Used load: 3 N Sliding frequency: 1 Hz	For both alloys, under dry condition, adhesive and abrasive wear occurred. Abrasive wear dominated the dry condition.	[88]
Stainless steel	Wrought	Micro-scale abrasion testing with artificial saliva mixed with abrasive particle. Used load range: 0.5 to 4 N Sliding velocity 150 rpm	With increasing load, the micro-abrasion rate increases. Ridge-dominated 2-body wear mechanism occurred at higher load.	[140]
Ti-6Al-4V, yttria-stabilized zirconia and zirconia toughened alumina	Yttria-stabilized zirconia and zirconia toughened alumina: powder sintering and hydraulic pressing	Pin-on-disc reciprocating tribo experiment under artificial saliva lubricated condition. Used load: 20 N load Sliding speed: 200 rpm	Zirconia-toughened alumina was better suited to resist material loss.	[199]
Cp-Ti and Ti-Cu alloy (Ti_x_Cu, x = 3, 7.1 and 12 wt %)	Conventional powder metallurgy compaction: Hidruded-dehidruded (HDH) technique	Ball-on-disk tribometer with integrated electrochemical cell with artificial saliva. Used loads: 1, 5 and 10 N Rotational speed 60 rpm	Increasing Cu content in the alloy results in eutectoid formation along the grain boundary and increases hardness and material loss due to wear also reduced. Cp-Ti experienced plastic deformation, while third bodies and larger debris particle dominated Ti alloy with higher Cu content.	[200]
AISI 304 L stainless steel	Wrought	Pin-on-disc tribo experiment in hank biological solution. Used load: 3 N and 5 N Sliding speed: 120 rpm	Bigger wear track and surface crack formed for higher load.	[201]
Ti-6Al-4V	Cast and powder compaction sintering	Ball-on-flat type tribo experiemnt in Fusayama Meyer artificial saliva solution. Used load: 5 N Sliding: speed 60 rpm	Along with predominant adhesive wear, isolated wear debris was also observed.	[75]
Ti_20_Nb_13_Zr Water-cooled (WC) Air-cooled (AC) Furnace-cooled (FC) Aged	Spark plasma sintering	Ball-on-flat reciprocating tribo experiment in artificial saliva lubricated condition. Used load: 10 N Reciprocating frequency: 5 Hz	Primary wear mechanism was abrasive. Trapped debris contributed as ploughing component. Oxide film formation was on air-cooled and aged samples.	[202]
